# Pharmaceutically active micropollutants: origin, hazards and removal

**DOI:** 10.3389/fmicb.2024.1339469

**Published:** 2024-02-14

**Authors:** Anuradha Gupta, Sandeep Kumar, Yashi Bajpai, Kavita Chaturvedi, Parul Johri, Rajesh K. Tiwari, V. Vivekanand, Mala Trivedi

**Affiliations:** ^1^Flavin Labs Private Limited, Lucknow, Uttar Pradesh, India; ^2^J. Somaiya College of Science and Commerce, Mumbai, India; ^3^Amity Institute of Biotechnology, Amity University Uttar Pradesh, Lucknow Campus, Lucknow, Uttar Pradesh, India; ^4^ICAR-Central Institute for Subtropical Horticulture, Lucknow, Uttar Pradesh, India; ^5^Bundelkhand University, Jhansi, Uttar Pradesh, India; ^6^Department of Biotechnology, AITH, Kanpur, Uttar Pradesh, India; ^7^Department of Biotechnology, MNIT, Jaipur, Rajasthan, India

**Keywords:** pharmaceutically active micropollutants, wastewater treatment plant, antibiotic resistance genes, remediation, potential threat

## Abstract

Pharmaceuticals, recognized for their life-saving potential, have emerged as a concerning class of micropollutants in the environment. Even at minute concentrations, chronic exposure poses a significant threat to ecosystems. Various pharmaceutically active micropollutants (PhAMP), including antibiotics, analgesics, and hormones, have been detected in underground waters, surface waters, seawater, sewage treatment plants, soils, and activated sludges due to the absence of standardized regulations on pharmaceutical discharge. Prolonged exposureof hospital waste and sewage treatment facilities is linked to the presence of antibiotic-resistant bacteria. Conventional water treatment methods prove ineffective, prompting the use of alternative techniques like photolysis, reverse osmosis, UV-degradation, bio-degradation, and nano-filtration. However, commercial implementation faces challenges such as incomplete removal, toxic sludge generation, high costs, and the need for skilled personnel. Research gaps include the need to comprehensively identify and understand various types of pharmaceutically active micropollutants, investigate their long-term ecological impact, develop more sensitive monitoring techniques, and explore integrated treatment approaches. Additionally, there is a gap in understanding the socio-economic implications of pharmaceutical pollution and the efficacy of public awareness campaigns. Future research should delve into alternative strategies like phagotherapy, vaccines, and natural substance substitutes to address the escalating threat of pharmaceutical pollution.

## 1 Introduction

Pharmaceuticals, considered one of the greatest milestones in development of science for mankind, have expanded the life spans, cured many from lethal ailments and greatly refined the standards of our lives. This milestone has become a serious environmental threat, and pharmaceuticals are now considered rapidly growing environmental pollutants (Patel et al., 2019). Since past three decades, it has been found that pharmaceutical residues are present in almost all ecological bodies, such as surface water (lake, river, stream and water from sea), under groundwater, water from treatment plant, effluents and influents (Hanif et al., 2020).

Pharmaceutically active micropollutants (PhAMPs) are used in agricultural, medicinal and biotechnological field. These substances include hormones, antibiotics and drugs. Major sources of PhAMPs in the environment come from human and veterinary applications and other sources are the excretion and release into the surroundings by sewage treatment plants (STPs; El-Fattah et al., 2023).

The global usage of pharmaceutically active compound (PhACs) has been estimated to approximately 100,000 tons and beyond per year (Jayasiri et al., 2013). Some of these PhACs are employed immensely as non-prescriptive medications and after consumption, they get excreted with urinal discharge and feces as metabolites. Global consumption of antibiotics has surged with the rate of 30 percent in the last 20 years, more than half of which is due to inappropriate use, resulting in large amounts getting released into our ecosystem (Kraemer et al., 2019). Prescence of PhACs in ecosystems in higher concentration contributed to health risks for land as well as water ecosystem including humans.

Present setup of Waste-Water Treatment Plants (WWTPs) are not fabricated in a way to eliminate such pollutants. This is because pharmaceuticals have poor biodegradability and good hydrophilicity, it becomes difficult to get-rid of them from water using old techniques for wastewater treatment. Also, they interact with micro- plastics often present in water at the surface which act as a carrier of pharmaceuticals in water system via π–π interaction, due to their hydrophobic/hydrophilic nature (Koelmans et al., 2022). The usual processes employed in treating waste- water (from municipal sources) cannot remove these micro level pollutants. This results into mixing of the unused pharmaceuticals into surface water, which leads to negative impact on the aquatic ecosystem and may also hinder with the production of drinking water (Gao et al., 2021; Petrović et al., 2021; Daughton and Ternes, 2022; Pedersen and Pedersen, 2022; Jin et al., 2023; Li et al., 2023).

As the traditional treatment methods are not capable enough for the treatment and elimination of the micropollutants, other substitutive methods can be employed such as coagulation – flocculation, adsorption (on activated carbon), oxidation, filtration (membrane bioreactor), use of biocatalysts (isolated or as whole organisms like bacteria, fungi and algae) (Silva et al., 2019) and nanomaterials can help curbing the problem effectively (Bhuyan and Ahmaruzzaman, 2023; Sanjeev et al., 2023; Sigonya et al., 2023).

One of the important reasons, why there has been great concern regarding pharmaceutical products and compounds, is that they bring out biological effects. They are fabricated in a very stable ways, so that they can be stocked for long durations and readily consumed. They are lipophilic in nature so as to cross the host membrane, and to reach the site of action, especially the ones consumed via oral route, they must have resistance toward enzymes and must not be hydrolysable at lower pH and must be highly mobile in liquid system (Mert et al., 2018; Mauro et al., 2021).

By virtue of such characteristics, pharmaceutical compounds get bio-accumulated, making them persistent and recalcitrant in nature and may adversely affect our ecosystem (aquatic as well as terrestrial). It has also been reported that PhAMPs can also enter food chains.

## 2 Pharmaceutically active micropollutants in water

**Figure 1:**
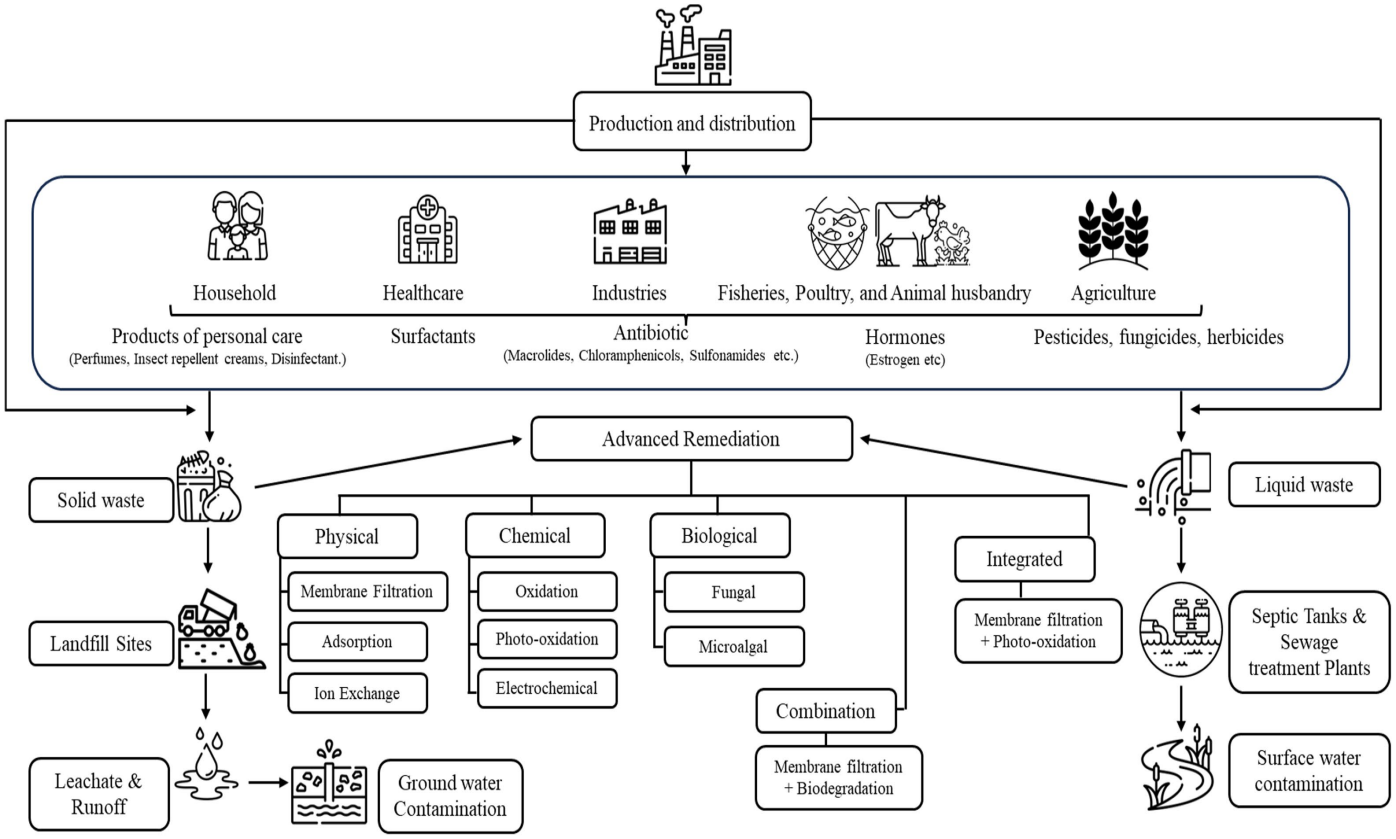
Remediation methods for pharmaceuticals and metabolites entering to waterbodies.

PhAMPs are usually present in water bodies in very trace amounts, in ranges of ngL^−1^ to μgL^−1^. Presence of PhAMPs in trace amounts and their heterogeneity convolute the detection and examination processes and pose hurdles for water treatment plants. Major sources of PhAMPs are shown in Table 1 and Figure 1.

**Table 1:** Categorization of pharmaceutically active micropollutants and their sources in the water bodies.

Category	Main sub classes	Sources	References
Pharmaceuticals	Antibiotics, NSAID^1^ Lipid modulator Stimulants Anticonvulsants	Wastewater from domestic households Effluents from health care facilities Pharmaceutical production plants Aquaculture & CAFO^2^	Liu et al. (2023a,b), Vadiraj et al. (2021), Mert et al. (2018), Karimi-Maleh et al. (2021), Bilal et al. (2019), Olicón-Hernández et al. (2017), Chaturvedi et al. (2021)
Products of personal care	Perfumes Insect repellent creams Disinfectant chemicals	Wastewater from domestic actions	Kumar et al. (2023), Chakraborty et al. (2023), Jyoti and Sinha (2023), Krishnan et al. (2021), Chaturvedi et al. (2021), Mert et al. (2018), Parra-Saldivar et al. (2021)
Steroidal hormones	estrogen	Wastewater from domestic actions Aquaculture & CAFO^2^	Narwal et al. (2023) Gubó et al. (2023) Chaturvedi et al. (2021), Krishnan et al. (2021), Mert et al. (2018), Parra et al. (2021), Vadiraj et al. (2021)
Surfactants	Non-ionic	Wastewater from domestic actions	Ratkievicius et al. (2023), Narwal et al. (2023), Mert et al. (2018), Vadiraj et al. (2021)
Chemicals and Pesticides	Plasticisers, Fungicides Fire extinguishers Insecticides, Herbicides	Industrial discharges Wastewater from domestic actions Agriculture	Annamalai et al. (2022), Parra et al. (2021), Mert et al. (2018), Krishnan et al. (2021), Vadiraj et al. (2021)

NSAID^1^, Non-steroidal anti-inflammatory drug.

CAFO^2^, Concentrated animal feeding operation.

## 3 Dispersal routes of pharmaceuticals in the surroundings

The excessive and improper prescription of antibiotics in human health and farming are associated with the abundance of antibiotics in the surroundings, especially in aquatic systems (Majumder et al., 2020). These actions result in the release of antimicrobials and/or in the form of metabolites in water bodies. This ultimately contributes toward surging antimicrobial resistance in microbes, by obtaining genes from the reservoir of Antibiotic Resistance Genes (ARGs) leading to their fast growth. It has been found that aquatic environments (primary habitats) and treatment plants act as a point for discharging a large chunk of pharmaceutical substances. Consequently, bacteria get optimal conditions where they develop and spread antibiotic resistance leading to the spread of ARGs. In this way, antibiotics and ARGs disseminate and get circulated between humans, fauna, and the environment. The secondary habitats include environments such as hospitals, nursing homes (where susceptible individuals with exposure to bacterial genetic exchange are present) and animal farms. The used pharmaceuticals or their metabolites from secondary habitats enter into wastewater treatment plants, where they get indulged with microbes (Table 2).

**Table 2:** Some commonly used pharmaceuticals and their origin.

Category	Pharmaceutical compound	Origin	References
Illicit drugs	α-methylphenethylamine (Amphetamine)	Urban and hospital wastewater	Mathur and Gehlot (2021), Olicón-Hernández et al. (2017), Al-Baldawi et al. (2021), Chaturvedi et al. (2021)
	N-methylamphetamine (Methamphetamine)		
Pharmaceuticals	Acetaminophen (Paracetamol)	Human intake and excretion in municipal wastewater, hospitals and pharmaceutical waste, and landfills	Liu et al. (2023a,b), Olicón et al. (2017), Chaturvedi et al. (2021), Krishnan et al. (2021), Sivaranjanee and Kumar (2021)
	Ciprofloxacin		
	Carbamazepine		
	Clofibric acid		
	Diclofenac		
	Diazepam		
Personal care products	Benzophenone	Domestic and industry effluent	Liu et al. (2023a,b), Sivaranjanee and Kumar (2021), Krishnan et al. (2021), Olicón et al. (2017), Al-Baldawi et al. (2021), Chaturvedi et al. (2021)
	Ethylhexyl methoxycinnamate		
	Galaxolide		
	Ethylhexyl methoxycinnamate		
Hormones	17-beta-estradiol	Human and veterinary treatment, hospital and domestic effluent	Sivaranjanee and Kumar (2021), Al-Baldawi et al. (2021), Olicón et al. (2017), Chaturvedi et al. (2021)
	Estriol		
	Estrona		

The sewage treatment centers being the tertiary niches, offer ideal environment for possible blending and genetic interchange between pharmaceutical compounds and the bacteria (Mutuku et al., 2022). Soil, sediments, surface and underground water provide the ultimate habitat wherein microbes from other habitats mingle and get associated with the larger bacterial groups in the existing habitat. This results in the interlinking of these niches, generating a point for breeding bacteria (antibiotic resistant) and ARGs, flowing in the environment and with time can re-enter into the terrestrial bodies. The regulation of the introduction of pharmaceutically active micropollutants into these habitats can be achieved by pre-treating hospital waste and creating awareness on consequences of inappropriate usage of anti-microbial agents.

## 4 Correlation between antibiotic contamination and resistance in the ecosystem

Studies have suggested that environments which are exposed to very high levels of antibiotic contamination show increased antibiotic resistance indicators (Tiwari et al., 2017). This can be well understood by an example of a study, wherein it was found that the ciprofloxacin concentration was present 1,000 times more than the inhibitory concentration for certain bacteria in the wastewater coming from a pharmaceutical manufacturing unit. It was also reported that there were more ARGs in the water going inside the Waste Water Treatment Plants (WWTPs) than the water coming outside. Another study reported multidrug-resistant bacteria isolated at the downstream of a river receiving oxytetracycline waste (Mutuku et al., 2022).

These findings have now clearly established the association between the plethora of ARGs and anti-microbial contamination in the environment and necessitates need for advancement in understanding the complex process and interactions involved in antibiotic resistance emerging from antibiotic contamination in the surroundings (Table 3).

**Table 3:** Antibiotics resistance genes (of the widely used antibiotics) found in the surroundings.

Antibiotic class	Antibiotic resistance genes	Environment	References
Macrolides	*mefA,phA*, *ereA2*, *mphB*, *ermA*, *ermO*, *ermF*, *ermB*	Water systems, influent, activated sludge, effluent	Wang et al. (2023), Bengtsson-Palme et al. (2016), Jiao et al. (2018), Sabri et al. (2020), Cheng et al. (2020), Wu et al. (2022)
Chloramphenicols	*catI*, *catII*	Natural water systems	Wu et al. (2022), Fu et al. (2023), Oberoi et al. (2019), Wang et al. (2023)
Trimethoprim and Sulfonamides	*dfrA*, *dfrB, sulI*, *sulII*, *sulIII*	Natural water systems, influent, activated sludge, effluent	Fu et al. (2023), Oberoi et al. (2019), Wu et al. (2022), Bengtsson-Palme et al. (2016), Jiao et al. (2018), Sabri et al. (2020)
Tetracycline	*tetA*, *tetA(C)*, *tetE*, *tetC*, *tetE*, *tetH*, *tetK*, *tetO*, *tetM*, *tetN*, *tetL*	Water from surface, sewage, fish pond, natural systems, activated sludges	Wu et al. (2022), Majumder et al. (2020), Mutuku et al. (2022), Cheng et al. (2020), Bengtsson-Palme et al. (2016), Zhang et al. (2016), Jiao et al. (2018), Wang et al. (2023)
β-lactams	*cit*,*ctx*, *ges*, *tem*, *shv*, *ampR*, *nps*, *sme*, *veb*	Different habitats, activated sludges	Wang et al. (2023), Li and Zhang (2011), Yang et al. (2013), Biswal et al. (2014), Amador et al. (2015), Oberoi et al. (2019)
Quinolone compounds	*gyr(A B)*, *qnrB*, *qnrC*, *qnrS, par(C, E), qnrA*,	Aquatic systems, Influent and effluent, activated sludge	Fu et al. (2023), Marti et al. (2013), Xu et al. (2015), Oberoi et al. (2019)
Glycopepetides	*vanA*, *vanD*, *vanC*, *vanB*	Aquatic systems	Oberoi et al. (2019), Wu et al. (2022), Fu et al. (2023)

## 5 Research concerns

The pharmaceutical industry continually introduces a significant number of pharmaceutical compounds to the market, and despite the fact that less than 2 percent have been detected in the environment, the production and release of pharmaceutically active micropollutants (PhAMPs) have increased alarmingly in recent years (Rovira-Alsina and Pijuan, 2018). This surge in PhAMPs is attributed to several factors, including the expansion of pharmaceutical usage, the persistence of these compounds in the environment, and the lack of effective regulatory measures to monitor and control their release (Bilal et al., 2022; Sharma et al., 2023).

Efforts to anticipate the concentration of PhAMPs in the environment involve intricate studies that consider various factors such as consumption rates, pharmaceutical usage patterns, the efficiency of wastewater treatment plants (WWTPs), and regulatory frameworks (Rovira-Alsina and Pijuan, 2018; Bilal et al., 2022; Sharma et al., 2023). However, predicting the precise environmental fate of PhAMPs remains challenging due to the complexity of their pathways and the influence of diverse environmental variables.

Adding complexity to the issue is the phenomenon of pharmaceutical metabolism, where a significant portion of the parent compounds undergoes transformation into metabolites known as transformation products (TPs). These TPs, often hydroxylated or conjugated forms, possess the potential to induce varied physiological responses and negative effects. Importantly, these metabolites persist and are released into untreated water at treatment plants, contributing to the overall environmental load of pharmaceuticals (Santos et al., 2015; Weber et al., 2021).

While traditional processing methods in WWTPs contribute to the natural inactivation of PhAMPs, they fall short of achieving complete removal for the majority of these compounds. This inadequacy has become a primary factor contributing to the discharge of micropollutants into surface water, leading to concerns about their impact on aquatic ecosystems (Li et al., 2022; Zhang et al., 2023).

To comprehensively address the environmental impact of PhAMPs, ongoing research is crucial. Researchers aim to unravel the intricate interplay between the production, release, and environmental fate of PhAMPs. This multidisciplinary approach encompasses studies on pharmacokinetics, environmental monitoring, and the development of advanced wastewater treatment strategies. Moreover, it facilitates the creation of more effective regulatory measures to monitor pharmaceutical production, usage, and disposal, ultimately mitigating the impact of PhAMPs on aquatic ecosystems. The evolving nature of this field underscores the necessity for continuous research to adapt strategies and regulations in response to the dynamic challenges posed by pharmaceutical pollution in aquatic environments.

## 6 Removal methods for pharmaceutically active micropollutants (PhAMPs)

In distinction with long-established wastewater treatment methods, recently developed methods for wastewater treatment, including membrane filtration, advanced oxidation processes (AOPs) activated carbon adsorption etc. have shown better removal potential for PhAMPs. Bio catalytic approaches are eco- friendly and sustainable substitutes offering lower energy inputs, newest reactions and producing few or nil harmful by-products than that generated by the traditional processes. Also, high enzyme specificity for their substrates reduces the possibility of undesirable reactions. Hence, using biocatalysts are said to be as an imminent choice to efficiently degrade micropollutants from water as well as wastewater (Table 4).

**Table 4:** Various methods used in removal of PhAMPs from water bodies.

Process	Sub categories	Examples	References
Physical segregation	Membrane filtration, Adsorption, Ion exchange, and Coagulation- flocculation	Reverse & forward osmosis, Nano-filtration, Zeoloite, Activated charcoal, Metal – organic systems, Anionic resin and cationic resin, Aluminum sulfate/Ferric cholride	Husain Khan et al. (2023), Noor et al. (2023), Majumder et al. (2020), Rovira (2018), Naghdi et al. (2018b), Mackuľak et al. (2021)
Chemical transformation	Chemical oxidation, Photo-oxidation, Electrochemical process	Ozonation, Fenton process, Photolysis, Photocatalysis, Photo –Fenton reaction, Electro-degradation, Electro-coagulation	Angeles-de Paz et al. (2023), Sahay et al. (2023), Kulišťáková (2023), Rovira (2018), Cai et al. (2018), Mackuľak et al. (2021)
Biological treatment	Biodegradation, Attached growth treatment	Micro-algal system, Fungal System, Biofiltration, Enzymatic Bioreactor	Chauhan et al. (2023), Bilal et al. (2022), Rovira et al. (2017), Robledo-Padilla et al. (2020), Jiménez-Bambague et al. (2020), Minggat et al. (2021), Wang et al. (2014), Mojiri et al. (2020), Xiong et al. (2018)
Combinatory treatment	Membrane filtration and biodegradation	Membrane bioreactor Membrane immobilized biocatalyst	Noor et al. (2023), Angeles-de Paz et al. (2023), Sahay et al. (2023), Mackuľak et al. (2021), Rovira (2018), Sahal et al. (2023)
Integrated process	Membranous filtration and Photo – oxidation	Membrane immobilized biocatalyst TiO2 immobilized biocatalyst	Sahal et al. (2023), Mackuľak et al. (2021), Rovira (2018)

### 6.1 Physical separation

Traditional wastewater treatment methods often struggle to remove PhACs, necessitating the exploration of advanced treatment technologies. Physical methods have emerged as promising tools for selectively removing PhACs from water, offering various advantages and challenges.

Several common physical methods are employed for the removal of pharmaceuticals and personal care products (PhACs) from water sources. Ion Exchange (IX) utilizes specially designed resins with charged functional groups to attract and bind PhACs with opposite charges. This method is known for its high selectivity and efficiency for specific PhACs and can be regenerated for reuse, contributing to its sustainability (Gómez-Serrano et al., 2017; García-Galán et al., 2018; Zhang et al., 2019; Mansouri et al., 2021). Another widely used method is Adsorption, where materials like activated carbon are employed to attract and retain PhACs on their surface. This method is effective for removing a diverse range of PhACs, however, it may require frequent regeneration or replacement of the adsorbent material to maintain its efficacy (Benner et al., 2020; Sharma et al., 2022). Membrane Filtration involves the use of semipermeable membranes with specific pore sizes to physically separate PhACs from water based on their size, with varying degrees of selectivity and effectiveness offered by different membrane types such as microfiltration and nanofiltration (Dolar et al., 2019; Nguyen and Chen, 2023). Coagulation and Flocculation, on the other hand, induce the aggregation of PhACs into larger particles through the addition of chemicals, facilitating their removal by sedimentation or filtration. While effective for larger-molecule PhACs, this method may not be as suitable for smaller or more soluble ones (Yu et al., 2020; Zhang X. et al., 2023).

Physical methods exhibit high removal efficiency, ease of operation, and simpler implementation, making them suitable for various water treatment applications. For instance, ion exchange not only demonstrates high removal efficiency but also offers the potential for regeneration and reuse, minimizing waste generation and enhancing its environmental friendliness. These characteristics collectively underscore the suitability of physical methods for the efficient removal of PhACs in diverse water treatment scenarios.

Several challenges are associated with the removal of pharmaceuticals and personal care products (PhACs) from wastewater using physical methods. The broad spectrum of PhACs present in wastewater, each with different properties, complicates the design of a single physical method capable of effectively removing all types of contaminants. The diversity in chemical structures and properties of PhACs necessitates a nuanced approach to address their varied characteristics. Moreover, the cost implications are substantial, especially for large-scale applications, considering the expenses associated with the procurement of materials and regeneration chemicals. Another challenge arises from competing ions present in the water, which can hinder the effectiveness of physical methods by vying with PhACs for binding sites on adsorbents or membrane pores. This competition diminishes the overall efficiency of the removal process. Additionally, the management of spent materials generated from adsorption or filtration processes poses a logistical challenge. Due to their concentrated PhAC content, these spent materials may require special handling or disposal methods to ensure environmental safety and regulatory compliance. Addressing these challenges is crucial for advancing the practical implementation and efficiency of physical methods in treating wastewater contaminated with PhACs.

Recent research has been dedicated to enhancing the performance and sustainability of physical methods for the removal of PhACs. Key strategies in this pursuit involve the exploration of new materials, with a focus on novel adsorbents exhibiting higher affinity and selectivity for specific PhACs, thus improving overall removal efficiency (Chen et al., 2022; Xu et al., 2022; Huang et al., 2023). Additionally, there is a growing emphasis on combining different physical methods within a multi-stage treatment process to provide a more comprehensive approach that can effectively address the diverse range of PhACs present in water sources (Zhao et al., 2021; Wang et al., 2022; Zhang et al., 2023). Efforts are also directed toward improving the regeneration processes for adsorbents and resins, aiming for enhanced efficiency and environmental friendliness to reduce operational costs and waste generation. Another avenue of research focuses on advanced membrane technologies, investigating new membranes with improved selectivity and antifouling properties. These advancements aim to enhance the overall efficiency and applicability of membrane filtration for the removal of PhACs, contributing to the ongoing refinement of physical methods (Ahmad and Bhatti, 2021; Jiang et al., 2022; Du et al., 2023). Collectively, these research directions signify a commitment to advancing the capabilities of physical methods, aiming for higher performance, sustainability, and broader applicability in addressing the challenges posed by pharmaceutical and personal care pollutants in water treatment processes.

Physical methods offer valuable tools for removing PhACs from water, providing high removal efficiency, ease of operation, and potential for environmental sustainability. Ongoing research and development efforts aim to address the existing challenges and improve the performance of these methods to create a more comprehensive and sustainable approach to managing PhAC contamination in water resources. While physical methods can eliminate large-molecule organic pollutants and Produce high-quality water, they struggle with pharmaceuticals and personal care products (PhACs) due to their inherent water solubility. Only a few PhACs, like diclofenac with its higher polarity, are partially removed by physical means. To effectively address PhAC contamination, physical methods should be combined with other approaches. This requires integrating complementary techniques like chemical or organic removal or implementing waste disposal systems. Such synergistic strategies ensure a comprehensive and tailored approach to water purification, considering the unique challenges posed by PhACs and other micropollutants.

### 6.2 Chemical transformation

Common chemical methods include Advanced Oxidation Processes (AOPs), employing highly reactive species like hydroxyl radicals to oxidize and decompose PhACs. AOPs comprise ozonation, Fenton and photo-Fenton processes, and photocatalysis (Gao et al., 2020; Sharma et al., 2021; Zhang et al., 2023). Chlorination, utilizing chlorine as a disinfectant, offers another method, though its effectiveness varies for different PhACs and may lead to the formation of potentially harmful disinfection byproducts (He et al., 2019; Yang et al., 2022). Chemical precipitation involves adding chemicals to form insoluble precipitates encapsulating and removing PhACs, with effectiveness dependent on specific PhACs and chosen chemicals (Li et al., 2019; Zheng et al., 2020). Sonochemistry, utilizing high-frequency sound waves to generate cavitation bubbles, facilitates the violent collapse of these bubbles, creating intense localized heat and pressure that can degrade PhACs (Ghauch et al., 2020; Wang et al., 2022).

Chemical methods boast high efficiency, often exceeding 90% removal, and broad applicability to diverse water sources. They can be adapted for wastewater effluents, surface water, and groundwater treatment. Additionally, some chemical methods offer targeted degradation, selectively degrading specific PhACs while minimizing the impact on other water constituents.

However, challenges accompany these chemical methods, including the potential formation of harmful byproducts, high energy consumption for processes like AOPs and sonochemistry, and the need for careful chemical handling and disposal to ensure environmental safety. Residual toxicity of some degradation products necessitates additional treatment steps.

Recent research endeavors focus on enhancing the efficiency, selectivity, and sustainability of chemical methods for PhAC removal. These include the exploration of new catalysts for AOPs, investigation into selective degradation methods that target specific PhACs while minimizing impacts on other constituents, integration of chemical methods with other treatment technologies for a comprehensive approach, and the utilization of solar energy to drive AOPs, thereby reducing their energy footprint and enhancing sustainability (References). These ongoing efforts underscore a commitment to refining chemical methods for the removal of PhACs, ensuring both efficacy and environmental responsibility in water treatment practices. Chemical methods offer a promising approach for removing PhACs from water due to their high efficiency, broad applicability, and potential for targeted degradation. Ongoing research and development efforts aim to address the existing challenges and enhance the performance of these methods to create a more sustainable and efficient solution for PhAC contamination in water resources.

### 6.3 Biological methods

Biological methods for the removal of Pharmaceutically Active Micropollutants (PhAMPs) represent a sustainable and environmentally friendly approach to mitigate the presence of these emerging contaminants in water and wastewater. These methods harness the power of various microorganisms, such as bacteria, algae, and fungi, to biodegrade or transform PhAMPs into less harmful compounds. One of the primary biological techniques is biodegradation, where microorganisms metabolize the PhAMPs, breaking them down into simpler, non-toxic substances. In some cases, these microorganisms can be naturally occurring in wastewater treatment systems, or specific strains can be engineered for enhanced performance. Another promising approach involves the use of phytoremediation, where aquatic plants are utilized to absorb and accumulate PhAMPs, which can then be harvested or subjected to further treatment. Biological treatment methods, however, may have limitations, such as the selectivity of microorganisms and the need for specific conditions to optimize their performance. Nevertheless, they offer a sustainable solution for the removal of PhAMPs and are increasingly being integrated into water treatment strategies to safeguard water quality and the environment.

#### 6.3.1 Fungal system

The utilization of fungal systems for biodegradation is emerging as a promising non-traditional approach in sewage treatment to eliminate micropollutants. These micropollutants encompass a variety of undesirable substances, such as pharmaceuticals and personal care products, as well as endocrine-disrupting compounds, contributing to rising environmental perturbations. Fungal systems, particularly those employing non-specific lignin-degrading enzymes, exhibit remarkable capabilities in removing a diverse array of complex pollutants commonly found in industrial effluents, including xenobiotics.

Fungal species belonging to the orders Polyporales and Agaricales within the division Basidiomycota and class Agaricomycetes are prominently featured in the removal of PhACs and other micro pollutants. Within the order Polyporales, species like *Pycnoporus sanguineus, Ganoderma lucidum, Trametes versicolor*, and *Irpex lacteus* are widely recognized for their effectiveness in biodegrading micropollutants (Zhu et al., 2021; Guo et al., 2022; Kumar et al., 2022; Liu et al., 2023a,b). Additionally, within the order Agaricales, species such as *Gymnopilus luteofolius*, *Stropharia rugosoannulata*, Pleurotus ostreatus, and *Agrocybe erebia*, although less commonly employed, also demonstrate potential in the biodegradation of micropollutants (Rovira et al., 2017).

Moreover, the application of whole fungal cells, specifically white rot fungi, and enzymes derived from fungi, including laccase, lignin peroxidase, and tyrosinase, further enhances the efficacy of fungal-based bioremediation processes for PhACs (Naghdi et al., 2018a). These enzymes play a crucial role in breaking down and transforming complex organic pollutants into less harmful substances, contributing to the overall success of the biodegradation process (Shahid et al., 2021; Duan et al., 2022; Morin-Crini et al., 2022; Li et al., 2023; Zhang et al., 2023).

The integration of fungal systems in sewage treatment not only signifies a shift toward environmental friendly and sustainable practices but also demonstrates the versatility of these organisms in tackling the challenges posed by emerging micropollutants. Ongoing research in this field continues to explore novel fungal strains, optimize biodegradation conditions, and assess the long-term environmental impacts, paving the way for the broader adoption of fungal-based biodegradation in sewage treatment practices.

#### 6.3.2 Microalgal system

Robledo-Padilla et al. (2020) have reported utilization of various freshwater or marine microalgae in the degradation of organic compounds. Jiménez-Bambague et al. (2020) showed that green microalgae removed up to 30–70% of pharmaceutical substances from household sewage water. The potential of *Chaetoceros muelleri*, a marine diatom to degrade pharmaceutical substances is not properly studied in earlier works. Minggat et al. (2021) put forward the use of *C. muelleri* in feeds used for aqua cultures as it grows rapidly and can be maintained easily. Wang et al. (2014) said that *C. muelleri* serves as a most satisfactory microalgae for lipid and biomass synthesis on a large scale. This microalgae species has also been used for elimination of macronutrients from waste water (Mojiri et al., 2020).

It has also been reported that microalgal cells can also bio adsorb the pharmaceuticals. Microalgae have adsorbed 0 to 16.7% of pharmaceuticals (like ibuprofen, metoprolol, paracetamol, estrone, diclofenac, trimethoprim, b-estradiol carbamazepine, and ethinylestradiol). Also, dead biomass of *Chlorella pyrenoidosa* and *Scenedesmus obliquus* adsorbed approximately 10 percent of the norgestrel & progesterone. Microalgae can also breakdown PhAMPs including caffeine, carbamazepine, tris (2-chloroethyl) phosphate, & ibuprofen. Irrespective of the advantages of this technology, researchers have also observed that high concentration of PhAMPs have negative effects on microalgae, such as changes in their chlorophyll content, carotenoid and protein content (Xiong et al., 2018).

### 6.4 Combinatory treatment

Combinatory treatment, which involves the synergistic use of both membrane filtration and bio-degradation techniques, presents a highly effective and comprehensive approach for the removal of Pharmaceutically Active Micropollutants (PhAMPs) from water and wastewater. This strategy takes advantage of the strengths of each method to address the unique challenges associated with PhAMP removal. Membrane filtration, typically using microfiltration, ultrafiltration, or nanofiltration membranes, is a well-established and efficient technique for physically separating suspended particles, colloids, and dissolved substances from water. It can effectively remove a wide range of contaminants, including PhAMPs, due to its ability to block or trap them based on size and molecular weight. Membrane filtration acts as a robust initial barrier, preventing PhAMPs from entering the treated water stream, and is particularly effective in eliminating suspended and colloidal matter. Bio-degradation, on the other hand, harnesses the power of microorganisms to biologically break down complex organic compounds, such as PhAMPs, into simpler, less harmful substances. This process can target a broader spectrum of contaminants, including those that may not be effectively removed by membrane filtration alone. By introducing specific microorganisms or enhancing the activity of naturally occurring ones, PhAMPs can be transformed into non-toxic by-products through metabolic processes.

When these two methods are combined, the membrane filtration step serves as a pre-treatment process that removes larger particles and physically blocks PhAMPs, ensuring that the subsequent bio-degradation step encounters a cleaner and less contaminated water stream. This enhances the efficiency and longevity of the biological treatment and minimizes fouling of the bioreactor, as the presence of particulate matter can impede microbial activity. Furthermore, membrane filtration also aids in preventing the release of potentially harmful by-products generated during bio-degradation into the treated water (Rovira, 2018; Mackuľak et al., 2021; Angeles-de Paz et al., 2023; Noor et al., 2023; Sahal et al., 2023; Sahay et al., 2023). Combinatory treatment for PhAMP removal is highly versatile and adaptable to various water treatment scenarios. It offers a robust and reliable solution for addressing the challenges posed by these persistent contaminants, helping to improve water quality and safeguard the environment while ensuring the efficient operation of water treatment systems. However, it’s important to consider factors such as membrane selection, the choice of microorganisms, and system design to optimize the performance of this integrated approach in specific applications.

### 6.5 Integrated process

The integrated process that combines membrane filtration with photo-oxidation is a highly effective and versatile approach for the removal of Pharmaceutically Active Micropollutants (PhAMPs) from water and wastewater. This two-step treatment strategy takes advantage of the unique strengths of both membrane filtration and photo-oxidation to comprehensively address the presence of PhAMPs in water sources. Membrane filtration, using microfiltration, ultrafiltration, or nanofiltration membranes, is an established and efficient method for physically separating suspended solids, colloids, and dissolved substances from water. This initial step effectively removes larger particles and micropollutants, including PhAMPs, by blocking or trapping them based on size and molecular weight. It acts as a robust barrier, preventing these contaminants from entering the treated water stream and ensuring a cleaner feed for the subsequent treatment.

Photo-oxidation, on the other hand, relies on the use of UV (ultraviolet) light or other advanced oxidation processes (AOPs) to break down complex organic compounds, such as PhAMPs, into simpler, non-toxic substances. During this step, the energy from the UV light initiates chemical reactions that result in the degradation of PhAMP molecules. Photo-oxidation can be highly effective in breaking down a broad spectrum of contaminants, even those that may be resistant to biological treatment methods.

The integrated process of membrane filtration and photo-oxidation is flexible and adaptable to various water treatment scenarios. It offers a highly efficient and sustainable solution for the removal of PhAMPs, with the potential to enhance water quality and ensure the reliable operation of water treatment systems. However, it’s essential to consider factors such as membrane selection, UV/AOP reactor design, and optimal operational parameters to achieve the best results in specific applications.

## 7 Summary

In summary, the detection of pharmaceuticals and their ecological impact is a relatively recent focus, with technology emerging in the last few decades despite earlier observations of their presence in ecosystems. While numerous studies exist on Pharmaceutical and Active Metabolite Products (PhAMPs), our understanding of their acute and chronic effects on humans, plants, and animals remains incomplete, and legal standards for maximum environmental concentrations of pharmaceuticals are largely absent, apart from isolated guidelines such as those in Australia. As conventional water treatment methods fail to effectively remove micropollutants, there is an urgent need for advanced tertiary treatment processes, particularly given population growth and increased *per capita* drug use. Techniques like Advanced Oxidation Processes (AOPs) and adsorption show promise in removing PhAMPs from water systems, although they can generate potentially harmful by-products. While adsorption has advantages, the management of used adsorbents poses challenges. Microbial remediation offers hope for PhAMP degradation, but further exploration of the potential of bacteria, fungi, and algae is necessary. This topic is an active research area, especially considering the continuous introduction of new pharmaceuticals.

Therefore, it is essential to develop advanced detection methods, emphasize monitoring in developing nations, establish standardized limits for pharmaceuticals in wastewater and the environment, enforce strict regulations on pharmaceutical waste, promote sustainable pharmaceutical production, investigate chronic PhAMP exposure’s effects on ecosystems and human health, and equip wastewater treatment plants with cost-effective, environmentally viable remediation technologies to address this emerging environmental concern.

## 8 Conclusion

In conclusion, the contamination of the environment with pharmaceutical micropollutants presents a growing concern, as even minute concentrations can have significant ecological impacts. The lack of standardized monitoring frameworks has allowed these pollutants to proliferate in various environmental settings, further exacerbated by the development of antibiotic-resistant bacteria in ill-treated waste and sewage. Conventional water treatment methods are ineffective at removing these pollutants, leading to the exploration of alternative techniques. However, challenges such as incomplete elimination, toxic waste generation, high costs, and skilled labor requirements hinder their widespread adoption. To address this issue and mitigate the circulation of antibiotic resistance genes, future research must prioritize innovative approaches like phagotherapy, vaccines, and natural alternatives to reduce excessive antibiotic use.

## Author contributions

AG: Data curation, Writing – original draft. SK: Data curation, Formal analysis, Writing – original draft. YB: Methodology, Writing – original draft. KC: Data curation, Writing – review & editing. PJ: Methodology, Writing – review & editing. RT: Investigation, Writing – review & editing. VV: Resources, Writing – review & editing. MT: Investigation, Resources, Supervision, Writing – review & editing.
